# Motives and consequences of musculoskeletal injuries management at traditional bone setting centers rather than hospital orthopedic departments in Khartoum, Sudan 2020

**DOI:** 10.4314/ahs.v24i2.39

**Published:** 2024-06

**Authors:** Hatim Hamad, Dina Omer, Rana Abdelnabi, Abrar Abdelgaleel

**Affiliations:** 1 Omdurman Islamic University Faculty of Medicine and Health Sciences, Orthopaedic Department; 2 Omdurman Islamic University Faculty of Medicine and Health Sciences

**Keywords:** Traditional bone setter, complications, Orthopedics

## Abstract

**Background:**

Traditional bone setter (TBS) is a traditional practitioner of joint manipulation who lack proper training and formal education, therefore many complications and fracture morbidity has been reported in relation to TBS malpractice.

**Material and methodos:**

The goal of this study was to determine the motives and subsequent complications of seeking TBS rather than orthopedic doctors even though patients should seek urgent proper medical care. Data of this study has been drawn from observational descriptive cross-sectional combined hospitals and community based, multicenter study in Sudan.

**Results:**

(55.3%) of participants were at TBS centers and (44.7%) were at hospitals for a variety of reasons; (66.7%) of total participants have utilized TBS services as either 1st or 2nd intervention before or following hospital management and complications were reported in (22.3%) of total participants utilizing TBS services.

**Conclusion:**

The number of patients seeking TBS centers exceeded the number of patients seeking hospitals for musculoskeletal injuries management. Finally there was no association between the educational level, socioeconomic status and the first action taken By patient toward their injuries.

## Introduction

Traditional methods of dealing with musculoskeletal MS injuries have often been considered by a significant number of populations worldwide. Here in Sudan, despite the remarkable development and evident availability of modern orthopedic services, many patients still choose traditional methods resulting in catastrophic complications and disabilities. Traditional bone setting is a highly specialized form of alternative medicine that deals with joint manipulation and musculoskeletal injuries management. In Sudan a traditional bone setter (TBS) is called Baseer, his name means a person who has vision beyond other people, TBS educate themselves from traditions and take up the practice of healing without having any formal training in accepted medical procedures^1^. There might be stories of patients who are treated successfully by a TBS but those who seek orthopedic treatment after consulting TBS do so due to complications resulting from TBS mismanagement. These complications range from minor limb discrepancies (caused by mal union of fracture) with minimal effect on function to major ones like limb gangrene and death^2^,^3^. The practice is usually preserved as family practice with the usual scenario that it is passed from father to son or Training via apprenticeship by dependence on experience and spiritual intuition^2^ promoting inheritance of improper knowledge of disease prevention and control measures. Unfortunately studies and researches regarding these problems are few.

## Justification

To shed light on the complications arising from TBS practice hence they lack proper training and formal education alongside refusing to be taught. And to highlight another well-known issue in Sudanese community regarding patronization of TBS despite their well-known shortcoming and availability of hospitals, and hopefully pave the groundwork for solving an eminent problem burdening Sudanese community.

## Aim

To determine the motives and consequences of seeking TBS treatment over orthopedic treatment at hospitals for Musculoskeletal injuries in Khartoum, Sudan.

## Objectives

1) To determine the motives of seeking TBS treatment over hospital orthopedic treatment for MS injuries.
2) To estimate the prevalence of MS injuries patients seeking TBS treatment first followed by hospital orthopedic treatment and likewise, vice versa.
3) Compare the effects of demographic factors such as education and socioeconomic status on their tendency to seek TBS treatment in MS injuries.
4) Compare the effects of first action taken by patients with MS injuries and them seeking either TBS or orthopedic hospital treatment.

## Methodology

### Study design and area

This is an observational descriptive, cross-sectional, combined hospital and community based multicenter study. Three hospitals (Omdurman teaching hospital, Ibrahim Malik hospital, Khartoum north teaching hospital) and three TBS centers in Khartoum, Sudan from January 2019 to January 2020.

### Study population

All musculoskeletal injured patients presented to orthopedic or TBS centers during time of data collection.

### Inclusion criteria

MS injured patients presented to traditional bone setting or orthopedic centers during time of data collection, had mental capacity to participate and agree to be included

### Exclusion criteria

MS injured patients who expressed complications developed after consulting orthopedic doctors, had proper follow up, and never visited a traditional bone setter.

### Sample size

Sample size (n) of unknown population. Where z= normal score= 1.96 for 95% confidence level

P= probability= 0.5

n = (z-score)^2 * p*(1-p)/(margin of error)^2

n = 3.8416*0.25/0.0025

Minimum n =384.16

### Statistical analysis

Data was analyzed using SPSS v20.20. , cross-tabulation used to demonstrate different variables association using chi-square statistics.

### Ethical considerations

Ethical clearance from the ministry of health was obtained. Permission to interview with patients was obtained from both hospitals and TBS. Informed verbal and/or written consents was taken from all participants or from their guardians' in case of patients were below 10 years old or disabled geriatrics.

Data was collected using a structured questionnaire in an interview mode. The variables of interest included in the variables of interest included the background characteristics of participants, Type of trauma, Type of injury, body part involved and hand dominancy, First step or action taken by the patient toward his/her injury, Reasons of seeking TBS management as first or second action, Reasons of seeking orthopedic management as first or second action, Type of intervention done, Complications that developed from TBS management and Intention of seeking Legal rights against complications inflicted on patients due to TBS management.

## Results

This study included a total of 389 patients. (55.3%) of which were at TBS centers, (44.7%) were at hospitals in time of data collection, (53.2%) were males, mean age group was (31-40) years and (33.7%) were educated until primary level . (table 1)

**Table 1:** background characteristics of participants:

Characteristics	Frequency n (%)	Total n (%)
**Age**		
less than 10	51 (13.1)	
10-20	65 (16.7)	
21-30	61 (15.7)	
31-40	69 (17.7)	
41-50	59 (15.2)	
51-60	39 (10.0)	
more than 60	45 (11.6)	389 (100.0)
**Sex**		
Female	182 (46.8)	
Male	207 (53.2)	389 (100.0)
**Marital status**		
Married	207 (53.2)	
Single	111 (28.5)	
Separated	9 (2.3)	
Widowed	11 (2.8)	
Underage (less than 10years old)	51 (13.1)	389 (100.0)
**Educational level**		
Primary	131 (33.7)	
Secondary	90 (23.1)	
Tertiary	80 (20.6)	
non-school educated	21 (5.4)	
None	67 (17.2)	389 (100.0)
**Occupation**		
housewife	83 (21.3)	
None	140 (36.0)	
manual workers	140 (36.0)	
High educated job	26 (6.7)	389 (100.0)

**Figure 1:**
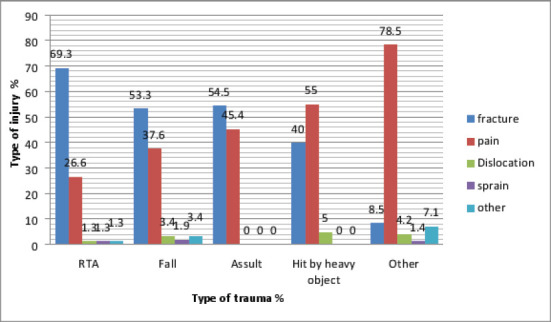
distribution of study population according to the type of injury in relation to the cause of trauma

**Figure 2:**
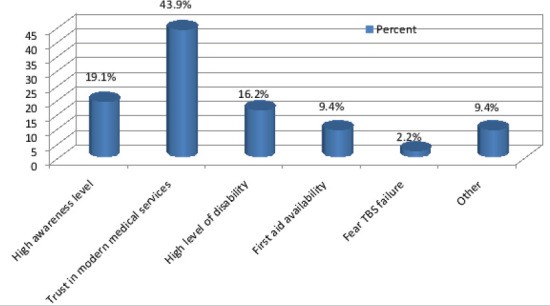
distribution of study population according to motives of seeking hospital as 1^st^ action

**Figure 3:**
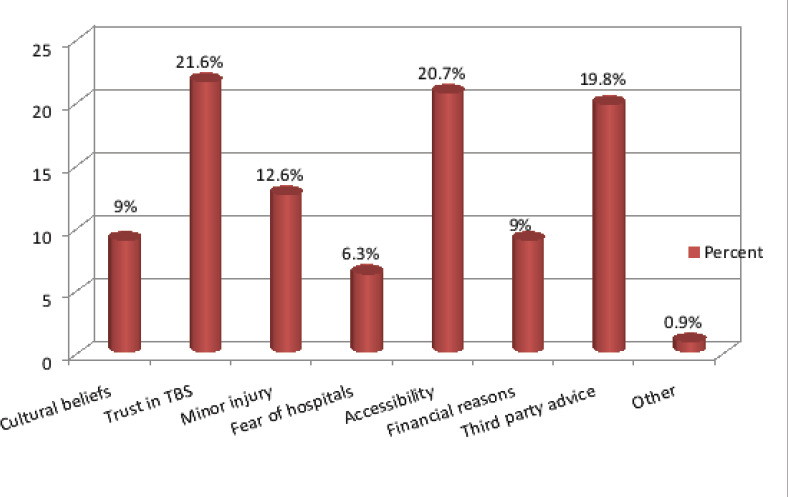
distribution of study population according to motives of seeking TBS as 1^st^ action

**Figure 4:**
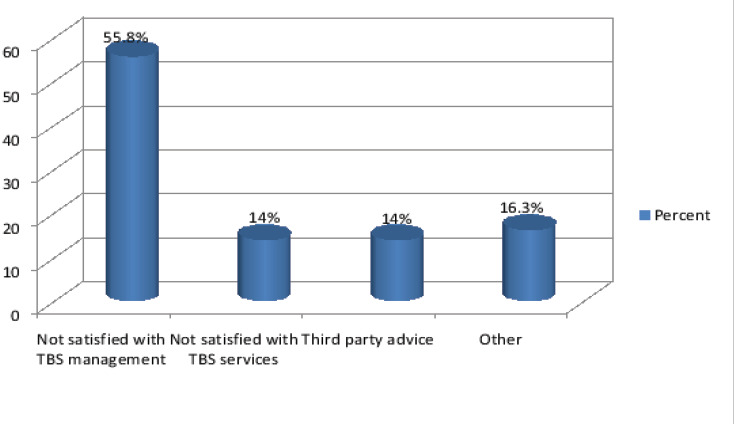
distribution of study population according to motives of seeking hospital after TBS

**Figure 5:**
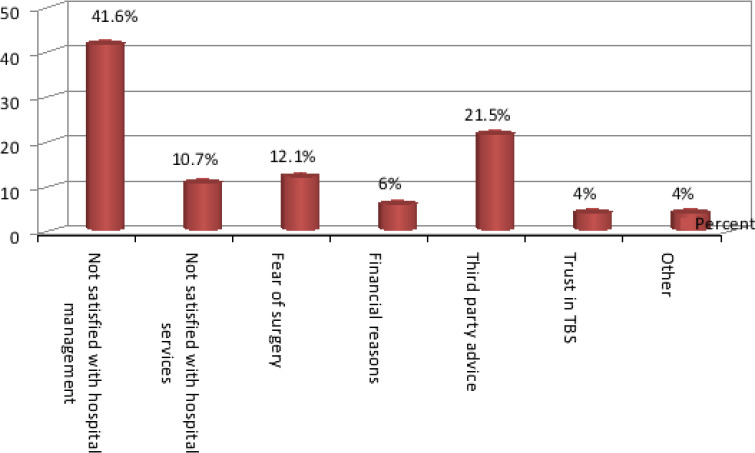
distribution of study population according to motives of seeking TBS after hospital

The commonest mechanism of injury was domestic falls (51.9%) and (47.8%) of injuries were fracture. The majority of cases were right upper limb injuries (25.7%) with (49.6%) had their dominant part involved (figure 1). (66.7%) of total participants utilized TBS services as either first or second intervention following hospital management. Patients who sought hospital first were (33.2%), trusting medical services was the most common cause (43.9%) (figure 2), also for patients who sought TBS their trust in TBS was the most common cause in (21.6%) (figure 3) of patients seeking TBS first representing (17.5%) of total number of patients. Eventually (11.6%) of patients sought hospitals after TBS, they were mainly driven by the unsatisfactory results of TBS management in (55.8%) (figure 4). 37.8 % of patients were seeking TBS after hospital visit (41.6%) of them sought a TBS after hospital visit because they were not satisfied with hospital management (figure 5).

There is a significant relationship between the type of injury and the sort of treatment chosen by patients as either first or second option P=.001 (Table 2). There is also a significant relationship between the level of education and occupation (which can be a significant determinant of socioeconomic status) with the initial sort of treatment that is being chosen by patients toward their injuries as (P=.005) , (P= .01) respectively (Table 3) TBS centers practiced Manipulation over (30.8%) of patients and traditional split (TAAB) over (20.1%). Conservative management (casting) accounted for (48%) in hospitals. Complications were reported in (22.3%) of total participants utilizing TBS services. The most frequent complication was loss of function in (21.9%) followed by deformity in (20.3%) (Table 4). (95.3%) of patients with complications refused to practice their legal rights against the complication inflicted on them by TBS.

**Table 2:** Relation between type of injury and sort of treatment n= 389(100.0%) P=.001

Sort of treatment					
Type of injury	Patients Seeking hospital first n(%)	Patients Seeking TBS first n(%)	Seeking Hospital after TBS visit n(%)	Seeking TBS after hospital visit n(%)	All n(%)
broken bone	80(43.0)	22(11.8)	28(15.1)	56(30.1)	186(100.0)
Pain	43(25.0)	40(23.3)	16(9.3)	73(42.4)	172(100.0)
Dislocation	0	3(25.0)	1(8.3)	8(66.7)	12(100.0)
Sprain	1(16.7)	1(16.7)	0	4(66.7)	6(100.0)
Other	5(38.5)	2(15.4)	0	6(46.2)	13(100.0)

Total	129(33.1)	68(17.4)	45(11.5)	147(37.7)	389(100.0)

**Table 3:** relation between patients educational level, occupation and chosen initial sort of treatment N=389 P=.005, P= .01

Initial sort of treatment				
Educational level	Seeking hospital care n(%)	Seeking TBS n(%)	Self-treatment at home n(%)	All n(%)
Primary	72(55.0)	32(24.4)	27(20.6)	131(100.0)
Secondary	50(55.6)	18(20.0)	22(24.4)	90(100.0)
Tertiary	59(73.8)	6(7.5)	15(18.8)	80(100.0)
Non-school	10(47.6)	8(38.1)	3(14.3)	21(100.0)
educated				
None	41(61.2)	20(29.9)	6(9.0)	67(100.0)
Total	232(59.6)	84(21.6)	73(18.8)	389(100.0)
occupation				
Housewife	35(42.2)	30(36.1)	18(21.7)	83(100.0)
Retired	1(33.3)	1(33.3)	1(33.3)	3(100.0)
Employee	24(75.0)	3(9.4)	5(15.6)	32(100.0)
Technician	3(100.0)	0	0	3(100.0)
Trades	17(63.0)	4(14.8)	6(22.2)	27(100.0)
Farmer/shepherd	4(44.4)	3(33.3)	2(22.2)	9(100.0)
Drivers	14(73.7)	2(10.5)	3(15.8)	19(100.0)
High educational	18(69.2)	0	8(30.8)	26(100.0)
jobs				
handyman	35(70.0)	7(14.0)	8(16.0)	50(100.0)
None	81(59.1)	34(24.8)	22(16.1)	137(100.0)
Total	232(59.6)	84(21.6)	73(18.8)	389(100.0)

**Table 4:** Relation between complications caused by TBS mal-practice and type of intervention of TBS. n=45

Type of intervention at TBS							
Complication caused by TBS	Manipulation	Herbs	Traditional Splints	Dry cupping	Massage	Other	Total n (%)
Deformity	3	0	1	0	1	8	13(20.3)
Non union	1	0	0	0	1	1	3(4.7)
Stiffness	3	0	0	1	0	0	4(6.2)
Swelling	3	0	3	0	1	3	10(15.6)
Local sepsis / infection	1	1	2	0	0	1	5(7.8)
Loss of	3	2	2	3	0	4	14(21.9)
function							
Mal union	0	0	1	0	0	0	1(1.6)
Re-fracture	2	0	0	0	0	0	2(3.1)
Neuropathy	0	1	2	0	2	2	7(10.9)
Other	0	1	1	1	0	2	5(7.8)
Total	16	5	12	5	5	21	64(100)

## Discussion

Sudan as a developing country full of traditional practices, TBS centers serve as point of contact to vast majority of population despite the availability of orthopedic surgeons unlike other countries as in Tanzania TBS services is considered as a critical source of management due to shortage of orthopedic surgeons^14^. This study shows the motives for seeking TBS and the consequences of this action. The main age group of patients presented with MS injuries were between 31- 40 years (17.7%), and the majority of patients were males (53.2%); Similar results represented by Kuubiere B. Callistus as (23.0%) of the subjects were within the 21-30 years old and males constituted about (67.8%)^3^. Patients in this study filled different educational levels, primary education (33.7%), secondary education (23.1%), tertiary education (university) (20.6%), none educated (17.2%) and (5.4%) for non-school educated, in relation to sort of treatment chosen by patients, as expected the relationship was positively significant for both relations of educational level and occupation (P=.005,P= .01) respectively for the initial sort of treatment chosen by patients. Evidencing that seeking TBS is a matter of believes more than illiteracy; in compare to other study by Dada A.A stated that patronization of traditional treatment is independent of educational status^12^. Most common etiology of trauma in this study was fall (51.9%) followed by road traffic accidents in (16.3%). Differing from Owumi, B.E study where a majority of the respondents indicated road accident as a major cause of fractures among injured individuals (85.2%)^7^.

In Aniekan Udoh Ekere study the most frequent cause was road traffic accidents (42.25%) followed by falls (30.99%) and sports injuries (9.86%)^2^. Most common type of injury was fractures (47.8%) and the diagnosis was confirmed by x-ray or MRI followed by pain (44.2%), others (3.3%) included abnormal function, limb loss and degenerative bone disease, dislocations (3.1%) and sprain (1.5%). In contrast to the study of Aniekan Udoh Ekere where fractures represented (86.05%) and dislocations (13.95%)^2^. Regarding anatomical distribution of involved body parts most of the injuries were upper limb injuries (49.1%) with right dominancy (84%) and left dominancy (11%), followed by lower limbs (39.1%), back (8%), neck (2.6%) and pelvis (1.3%), close to Aniekan Udoh Ekere study which revealed most of these injuries occurred in the upper extremity (43.02%) while lower extremity were (56.98%)^2^. The first action taken by patients toward their injuries was seeking hospital (59.6%), (21.6%) of them sought TBS and (18.8%) chose self-treatment at home initially. (55.3%) of participants were at TBS centers and (44.7%) were at hospitals and (66.7%) of total participants have utilized TBS services as either 1^st^ or 2^nd^ intervention before or following hosptal management. In compare to Aniekan Udoh Ekere study found that after injury (63.3%) consulted TBS first while (36.6%) consulted an orthopedic doctor^2^; Indicating that most of Musculoskeletal injured patients who have been interviewed ended up at TBS regardless the fact that they sought hospital first. As regard to the distribution among study population the major sort of follow up in this study was seeking TBS after hospital in (37.8%), seeking hospital first in (33.2%), seeking TBS first in (17.5%) and (11.6%) for seeking hospital after TBS; In comparison with Owumi, B.E study which resulted in that (33%) of the respondents visited hospitals before their withdrawal, while the remaining did not visit a hospital (67%)^7^, and Hamza Hassan Khan study that showed (29.5%) of the patients utilized alternative medicine for their primary complaint^6^.

Another study by Nardous W has shown that 29.9% of patients in Addis Ababa, Ethiopia preferred TBS over hospitals for management^13^. Motives behind patronization of TBS as first option was mainly for trust in TBS representing (21.6%). (20.7%) for accessibility, (19.8%) because of third party advice, (12.6%) thought it was a minor injury, (9%) for both cultural believes and financial reasons, (6.3%) for fearing of hospitals and (0.9%) for others which included avoiding of legal repercussions and for appease curiosity. Other study by Owumi, B.E resulted in reasons for non-visitation of a hospital when the injury occurred was (30.5%) for Unconsciousness, (25.4%) for Fear of amputation, (18.6%) no better service, (13.6%) nearer and (11.9%) for cheaper fees^7^. While seeking TBS as second option it was mainly for the unsatisfactory services of hospitals in (41.6%) (Receiving a conservative management only, patient condition didn't improve or developed complications, doctors failed to provide enough information). (21.5%) for third party advice, (12.1%) for fear of surgery, (10.7%) due to hospital system failure (long patient waiting, poor housekeeping, water damage), (6%) because of financial reasons and (4%) for both trust in TBS and others including misunderstanding of physical therapy advised by orthopedic doctor, fear of cast, and continued doctor strikes behind Sudan protests. Results were different from Owumi, B.E respondents response for the reason for withdrawal from the hospital were (79.3%) for no improvement, (13.8%) for fear of amputation and (6.9%) for quick services.^7^ Accordingly some of these motives can be considered reasonable as for those patients who utilized TBS for financial issues and easiness of access. whereas motives of seeking hospital first varied among patients but the main reason was trust in medical services in (43.9%), (19.1%) for high awareness level, (16.2%) for multiplicity of injuries after major trauma, (9.4%) for both first aid availability and others including TBS requesting x-ray, third party advice and (2.2%) for fear of TBS failure.

While seeking hospital as second option was mainly due to the unsatisfactory results of TBS management in (55.8%). (16.3%) due to others including TBS advice, development of new symptom other than primary complaint, (14%) for both not satisfied with TBS services and third party advice. Types of interventions done in hospital were (60%) for conservative management (i.e. cast, arm sling, splint), (24.6%) for operative management (i.e. internal fixation, external fixation), (15.4%) for others as pain relief medication, waiting for scheduled surgical intervention and physiotherapy. (11.6%) of total participants were receiving management at orthopedic centers after they were treated by a TBS, according to the sort of intervention they got at hospitals (27.8%) required surgical intervention, (39.5%) required casting, and only (11.6%) needed conservative management. In compare to a study by Aniekan U.E. the most frequent intervention was surgical management (open reduction and internal fixation 69%), followed by manipulation under anesthesia (9.86%)^2^. As regard to type of intervention of TBS in this study (30.8%) of patients were managed with manipulation, (20.1%) with traditional splint (TAAB), (19.6%) with massage, (14.5%) with dry cupping (Hijama) which is an act that is widely used by TBS without any cautions or care for sterilization, (12.1%) with herbs, some of the commonly used herpes was (commiphora myrrha, menthol, cinnamomum, camphora oil), and (2.8%) were given other options including vitamins, swimming advice, cauterization, or using a coin wrapped in plater then fixed to the clavicle at the site of injury claiming it plays a role in healing process. At TBS the process of making diagnosis relies on x-ray finding and physical assessment, unlike a similar study in Sudan by Ibrahim A. E. O that showed TBS diagnosis is carried out Without the benefit of anatomical dissections or x-ray photographs^4^. Nevertheless the manipulation was considered to be a leading cause of complications n=16 (35%) as there was adopted ways away from readily discernable scientific basis. After manipulation (TAAB) which is a traditional splint made of aligned wooden sticks in a cross tied shape being applied over injured limb. Splinting materials are wrapped into mat-like splint and applied tightly over the fracture site without padding along with the herbal mixture, sometimes joint above and joint below are immobilized. Traditional splints is regularly related to traditional healers among cultures even though it may differ in its component as in South Australia it was made of clay and Rawhide^1^. The Shoshone Indians soaked strips of fresh rawhide in water and wrapped them around limbs. Rawhide and clay hardened when it dried protecting the injured bone^1^. Treatment techniques and principles of bone setting may differ from a TBS to another. A study in Nigeria by Ekere and Echem revealed that Shortcomings of TBS healing methods are well-known in hospital practice^2^. Reported complications of TBS practice are due to lack of proper training and formal education^7^. In this study the most common resulted complications was loss of function (21.9%), deformity (20.3%), swelling (15.6%), neuropathy (10.9%), local sepsis and infection (7.8%), stiffness (6.1%), nonunion (4.7%), and re-fracture (3.1%); in compare to other study by Aniekan Udoh Ekere showed that the most frequent complication was nonunion in (36.4%)^2^ , mal union (24.71%) and both were associated with shortening in (31.76%). Other complications were chronic joint dislocation, ankylosis, joint stiffness, arthrosis/arthritis, chronic osteomyelitis, Volkmann's ischemic contracture, osteonecrosis, neuropathy, limb gangrene, delayed union and pressure ulcer^2^. As regard to the developed complications caused by TBS malpractice (95.3%) of patients refused to practice their legal right against TBS. Patients who refused mentioned that it is their fault that they sought TBS management. In Ethiopia a study demonstrated that 58% of patients seeking TBS were aware if its shortcoming^13^ also in northern parts of Ghana TBS enjoys a high level of acceptance, about 78% of all patients with fracture resort to TBS for treatment despite of the notwithstanding associated complications^3^.

Eventually patients with complications are referred to hospital for further treatment, their injuries became even more difficult to treat, costly and sometimes may requires amputation making people think that the only treatment option by orthopedic doctors is amputation^3^ all of which reinforce the fears of orthopedic treatment methods.

## Conclusion

By all count the total number of patients seeking TBS in Khartoum/Sudan exceeded the number of patients seeking hospitals for MS injuries management by a ratio of (1.2: 1). Most common represented group was patients seeking TBS after hospital visit, yet the number of patients seeking hospital as a first action is the greatest of all categories. There is an authentic trust given to TBS with regard to patient's educational level, socioeconomic status and/or type of injury.

## Limitations of study

This study had a number of potential limitations as in most descriptive studies. It was restricted by the measures used. The variables were filled with uncorrelatable facets which would have allowed more significant associations, and target groups responses were subjective, culturally inclined, and the phraseology might has influenced the finding. This study was cross-sectional in nature where we only assessed the respondents motives and consequences at a specific time(lack of follow up). Moreover the study was carried out in political and socio-economic unrest period where doctor strike, and unstable lifestyle were common which could've impacted the outcome of the study.

## References

[1] Singh P, Singh P, Bindra S (2013). Traditional bone setting: origin and practice. Int J Ther Appl.

[2] Ekere AU, Echem RC (2011). Complications of fracture and dislocation treatment by traditional bone setters: A private practice experience. Nigerian Health Journal.

[3] Kuubiere BC, Abass A, Mustapha I (2013). Fracture complications after treatment by traditional bone setters in Northern Ghana. Adv ApplSci Res.

[4] Ibrahim AE (2015). Knowledge, Attitude and practice towards Traditional Bone Setting in Fractures Management among Adults in SarasirVillage, ALhassahisa Locality, Gezira State, Sudan. Doctoral dissertation.

[5] Idris SA, Mohammed OB, Basheer ES (2010). Why do people prefer traditional bonesetters in Sudan?. Sudan Journal of Medical Sciences.

[6] Khan HH, Niazi AK, Ghazanfar H, Khan GH, Chaudhry MA, Khan ZH, Assad S, Khan LH, Qureshi MO, Orakzai SH (1970). Use of complementary and alternative medicine in orthopedic patients in Pakitan: A cross-sectional study. Rawal Medical Journal.

[7] Owumi BE, Taiwo PA, Olorunnisola AS (2013). Utilization of traditional bone-setters in the treatment of bone fracture in Ibadan North Local Government. International Journal of Humanities and Social Science Invention.

[8] Said GZ (2002). The management of skeletal injuries in ancient Egypt. AO Dialogue.

[9] Pettman E (2007). A history of manipulative therapy. Journal of Manual & Manipulative Therapy.

[10] Omololu AB, Ogunlade SO, Gopaldasani VK (2008). The practice of traditional bonesetting: training algorithm. Clinical Orthopaedics and Related Research.

[11] Debbarma J, Deb D, Deb S, Datta BK Traditional bone setting (TBS): An ethno-orthopedic healing practice of Tripura, NE India.

[12] Dada AA, Yinusa W, Giwa SO (2011). Review of the practice of traditional bone setting in Nigeria. African Health Sciences.

[13] Worku N, Tewelde T, Abdissa B, Merga H (2019). Preference of Traditional Bone Setting and associated factors among trauma patients with fracture at Black Lion Hospital in Addis Ababa, Ethiopia: institution based cross sectional study. BMC Research Notes.

[14] Card EB, Obayemi JE, Shirima O, Lazaro M, Massawe H, Stanifer JW, Premkumar A, Sheth NP (2020). Practices and perspectives of traditional bone setters in northern Tanzania. Annals of Global Health.

